# Social media use and body image issues among adolescents in a vulnerable Louisiana community

**DOI:** 10.3389/fpsyt.2022.1001336

**Published:** 2022-11-03

**Authors:** Caroline E. Sagrera, Johnette Magner, Jazzlynn Temple, Robert Lawrence, Timothy J. Magner, Victor J. Avila-Quintero, Pamela McPherson, Laura Lane Alderman, Mohammad Alfrad Nobel Bhuiyan, James C. Patterson, Kevin S. Murnane

**Affiliations:** ^1^Department of Psychiatry and Behavioral Medicine, School of Medicine, Louisiana State University Health Shreveport, Shreveport, LA, United States; ^2^Louisiana Addiction Research Center, Shreveport, LA, United States; ^3^School of Communication and Media Studies, Louisiana Tech University, Ruston, LA, United States; ^4^Caddo Parish Magnet High School, Shreveport, LA, United States; ^5^Child Study Center, Yale School of Medicine, New Haven, CT, United States; ^6^Division of Clinical Informatics, Department of Internal Medicine, Louisiana State University Health Shreveport, Shreveport, LA, United States; ^7^Department of Pharmacology, Toxicology and Neuroscience, School of Graduate Studies, Louisiana State University Health Shreveport, Shreveport, LA, United States

**Keywords:** Louisiana, social media, public health, adolescent, social determinants of health, disparities, body image

## Abstract

Widespread concern has been expressed regarding unrealistic body image and adolescent eating disorder promoting content on social media (SM) platforms. Numerous research studies have examined the impact of SM on body image as well as social vulnerability on negative mental health outcomes. Despite this, few previous studies have examined the impact of SM on body image specifically in vulnerable, underserved, or predominantly minority communities. This study examines the impact of SM on body image issues (BII) in adolescents in a public school system where greater than 50% of the students live in impoverished households. In late 2019, high school student leaders in Northwest Louisiana developed a survey alongside Step Forward, a collective impact initiative. Questions investigated adolescent SM use and mental health in Caddo Parish, namely BII. Teachers within Caddo Parish Public School System administered the survey. Out of the 11,248 total high school students in the school system, nearly 50% were sampled for a sample size of 5,070. Hypotheses included: (1) females were more likely to use SM than males, (2) increasing time spent on SM would correlate with females reporting BII, with males remaining largely unaffected, and (3) highly visual social media (HVSM) platforms would be associated with greater reports of BII than non-HVSM platforms. Results showed females were more likely to use SM (*p* < 0.001) and report BII (*p* < 0.001) compared to males, while both sexes reported BII with increasing time spent on SM (*p* < 0.001). A diversity of platforms were associated with increased BII among SM users compared to non-users (*p* < 0.001): Pinterest, Reddit, Snapchat, TikTok, Twitter, and YouTube. This conclusion is tempered by the omission of race as a variable in the study design, the use of self-report, and the use of an unvalidated instrument. These findings suggest that the harmful association between SM use and BII may transcend culture and socioeconomic status for a broadly deleterious effect on adolescent mental wellbeing.

## Introduction

### Adolescent vulnerability to mental health disorders

Previous studies have seen an association between heightened vulnerability to outside influences and the advent of puberty. Given the magnified sensitivity of this time, adolescents may be susceptible to mental health insults, with around 20% of children and adolescents experiencing some sort of struggle with mental wellbeing (1), and nearly 50% of those having developed signs and symptoms as early as 14 years of age (2, 3). Additionally, the prevalence of mood disorders increases with age, with rates nearly doubling from the age ranges of 13–14 to 17–18 years old (4). The most common mood disorders experienced by these populations are anxiety and depression, with nearly one in three adolescents suffering from some form of anxiety disorder. Furthermore, Kessler et al. found the prevalence of any diagnosable mental health or behavioral disorder in the adolescent population to be 23.4%, representing nearly a quarter of the entire adolescent population suffering mentally (2).

### Body image issues among adolescents

The phrase “body image” encompasses how one perceives, acts toward, thinks, and feels about one’s body and lies on a spectrum ranging from positive to negative perceptions (5). Adolescents, especially females, have historically been subjected to pressures in favor of the thin body image ideal in traditional media outlets; this problem is suspected of growing more severe with the rise of social media (SM) (6).

On average, 50% of adolescent females are unhappy with their bodies compared to 31% of males (7). Generally speaking, the media portrays several ideal body images for females, ranging from curvy and hourglass, to thin, to athletic and muscular (8–12). Meanwhile, males seem to receive a more consistent message that muscular is ideal (1).

### Impact of social media

Of all media outlets, SM may represent the strongest driver of these beauty ideals. SM platforms, such as Facebook, Instagram, Snapchat, and TikTok, among others, are used ubiquitously among U.S. adolescents, with 96% using at least one social networking platform (13, 14). There are differences between and among adolescents in their usage patterns, with 35% of adolescents of African descent indicating that they “enjoy using social media a lot” as opposed to 20% of adolescents of European descent. Adolescents of African descent also average 36% more time on SM compared to adolescents of European descent. In addition, in 2019 average daily screen use among adolescents of African Descent was 8.32 h compared to 6.40 h for adolescents of European descent (15).

SM platforms can be broadly divided into highly visual social media (HVSM) and others. HVSM includes Instagram, Snapchat, Pinterest, YouTube, and TikTok, among others in the minority. The “other” group is largely comprised of Facebook, Twitter, and Reddit. HVSM are often accompanied by interactive features such as likes, comments, and stories. McCrory et al., described these features as contributing to severe emotional highs and lows associated with instant gratification and instant inadequacy, respectively (16). It has been reported that females are disproportionately likely to use HVSM, with males tending to gravitate toward Facebook (17).

SM has enabled the widespread viewing of “ideal body image” content, with recent research indicating that up to 80% of photographs are digitally altered (18). Data from several SM platforms shows that they consistently reinforce these body image ideals and may facilitate poor body image, perception of self, and eating disorders among males and females (19, 20). Adolescents, especially females, have reported they received mostly negative insight about their bodies and perceived sexual attractiveness from SM, spurring body and self-dissatisfaction (21, 22). Furthermore, screen time on SM has been linked to obesity, perpetuating the unrealistic nature of the beauty standards portrayed on platforms. Body dissatisfaction has been strongly linked to associated symptoms of anxiety and depression, with data showing females at an increased risk, perhaps due to increased use of highly visual SM as compared to males (17, 23).

Additional studies examining time on SM have found that adolescents using SM for more than 2 h a day were more likely to report body image issues (BII), eating concerns, and depression (17, 24–26). This combination of time spent on SM and increased reliance on HVSM by females may serve as a potential reason why females appear to be more susceptible to BII compared to males (17).

### Caddo Parish population profile

These topics are especially relevant in the community of Northwest Louisiana, specifically Caddo Parish (county). Caddo Parish is located in the Northwest corner of Louisiana, bordering Texas and Arkansas. It is the fourth largest parish in Louisiana, containing three of the poorest zip codes in the state that are home to approximately 96,000 families. Caddo Parish is home to a population of about 240,000, comprised of 50% Americans of African descent, 46.5% Americans of European descent, 2.9% Hispanic Americans, and 1.3% Americans of Asian descent, with a median household income of $41,797 (27). Students of Caddo Parish Public Schools are comprised of 80% social minority populations, with 63.2% Americans of African descent, 32% Americans of European descent, and 4% of students from other races (28, 29). Furthermore, 54% of students in the school system are eligible to receive free and reduced lunch, an indicator of lower family income (30–32).

Depending on the year, Louisiana oscillates between ranking first and second nationwide in children living in poverty (33, 34). Caddo Parish is one of the most economically segregated regions in the South, with approximately 21% of families living below the poverty level (35). Childhood and adolescent adversity are often interrelated with poverty.

Louisiana ranked 50th among the states with the worst health outcomes in the United States (34). Louisiana ranks second in worst states for healthcare and ranks well above the national average for obesity and hypertension (34, 36–38). Specifically Shreveport, the largest city within Caddo Parish, ranks eighth in most obese cities nationwide (39). The state ranks 46th in public healthcare, and, finally, 49th in mental healthcare and mental health outcomes (34, 40).

These environmental factors expose the vulnerable adolescent population to significant stressors early in life, putting them at higher risk for physical harm and mental disability. According to the Child and Adolescent Health Initiative, 25.2% of Louisiana children had two or more adverse childhood experiences in 2016 (41). Studies show that exposure to extreme life stressors, such as childhood maltreatment, leave adolescents vulnerable to mood disorders, substance use disorders, impaired immune function, and generally poor health-seeking behaviors (42–44). These data portray a landscape ripe for mental health vulnerability and susceptibility to negative influences, highlighting the need for further data on other factors’ influence, such as SM, on adolescent mental health in areas with multi-generational poverty, violence, and social neglect (45).

In 2020, slightly more than 50% of Caddo Parish 10th grade students reported feeling depressed or sad most days in the past year and 35% met criteria for needing mental health treatment. This represents a 19% increase from the previous (2018) survey (1, 46).

### Field contribution

A National Library of Medicine electronic (PubMed) search conducted using the principal keywords—“social media,” “adolescents,” “body image,” and “disparities”— revealed four articles and a gap in the literature. Removing “disparities” resulted in numerous distinct results involving SM and BII in populations of young adults, Latinx, British, and Australian participants. However, no other study has assessed a population wherein the preponderance of children across an entire parish that is majority Americans of African descent or that ranks in the highest percentile for social vulnerability (47–50). Furthermore, this is the first study to examine the impact of BII in adolescents in a public school system where greater than 50% of the students live in impoverished households (31, 32, 51). Lastly, no previous studies have looked at impoverished adolescents with a sample size of such magnitude; the others of similar size focus on 18 years and older or neglect to account for social minority populations (50, 52–54).

### Hypotheses

We hypothesized that (1) females were more likely to use SM than males, (2) the more time spent on SM, the more likely females are to report BII, with males remaining largely unaffected, and (3) HVSM platforms are associated with greater reports of BII than non-HVSM platforms. These hypotheses may prove particularly acute for the disproportionately vulnerable adolescent community of Caddo Parish. With the onset of COVID-19, this conversation proves increasingly relevant. Our novel findings may set a foundation for future studies in these areas and inspire future early adolescent mental health interventions, especially as SM use rises.

## Materials and methods

### Study design

This study design was developed by students from the Caddo Parish Public School System who recognized the mental health crisis of their peers. These students felt SM to be a significant effector and perpetuator of BII and mental illness in their community. In response, during the spring of 2019, the Step Forward Teen Advisory Committee (TAC) was established to allow local adolescents to share their perspectives and knowledge of the needs of youth in Northwest Louisiana.

Step Forward represents a collective impact initiative of the Community Foundation of Northwest Louisiana. Their mission is to ensure optimal conditions of success for every individual from infancy to career in Northwest Louisiana. The TAC served as advisors to Step Forward community leaders in the process of identifying central focus areas and creating action plans geared toward improving student outcomes.

The TAC identified three key objectives for Northwest Louisiana youth: (1) improve teen mental health, (2) increase diverse career training opportunities, (3) increase youth civic involvement. In addition, the TAC wanted to understand the current state of adolescent mental health in Northwest Louisiana. Believing there may be a crisis with this population, they set out to collect data that accurately reflected the mental health landscape of Northwest Louisiana adolescents.

The TAC recommended specific question areas to Step Forward staff to create a survey instrument. The instrument consisted of 15 questions targeted at understanding teen mental wellbeing. Questions were asked about participants’ age and gender, use of SM, access to and utilization of support systems, various challenges experienced, coping strategies to stressors, and community involvement. Permission was obtained from the Superintendent of Schools for Caddo Parish for survey initiation.

The instrument designers felt it important to provide respondents with terms that were familiar with or would readily understand when answering the survey. With this in mind, more generalizable terms such as “issues with body image” were employed. This phrase was used to capture the landscape of adolescent BII more broadly and to “rule in” respondents who may be affected rather than create more narrow, specific criteria that have the potential to “rule out” due to respondent incomprehension or non-identification with an unfamiliar language.

### Data collection

During the 2019–2020 school year, there were 11,248 students enrolled in ten public high schools in Caddo Parish. The numbers of students per grade were as follows: 9th–3,156; 10th–2,802; 11th–2,666; 12th–2,624. Approximately 70% of Caddo students are economically disadvantaged with 71% identifying as a minority population and 29% identifying as white. Within the student population, 49.52% were female, and 50.48% were male. In the fall of 2019, the survey was available to English teachers in all 10 high schools in Caddo Parish to administer in their classes. Participation in the survey was voluntary, and parents were provided the opportunity to opt-out their children (55). Of the 11,248 high school students in Caddo Parish at the time of the survey, 5,070 respondents aged 14–19 years old, with a median age of 16, completed the survey. Because the survey was voluntary, participation was not tracked by the Caddo Parish Public Schools or school principals. We lack documentation of the specific schools and classes that participated in the survey. However, we believe that the volume of respondents is still sufficient to assign significance to the findings.

Later that year, TAC members worked with an expert consultant to analyze the data and provide a series of recommendations for improvement steps in students’ mental health services. These findings and recommendations were presented by Step Forward and TAC representatives to the Caddo Parish School Board.

Data obtained from this survey illustrates students’ beliefs regarding their own mental health. Topics of self-harm behavior, thoughts on confiding in someone beneficial, and BII were assessed. Analysis of this data allowed researchers to make inferences on various challenges faced by respondents, enabling the TAC team to better understand the mental health landscape of the Caddo Parish Public School system. At the beginning of 2020, Step Forward made this data set available to the broader research community with the intention of further data analysis for additional improvements in students’ mental health services. Our research team at Louisiana Health Sciences Center completed an IRB and received approval for study initiation.

We used the data available to give a cross-sectional view of adolescent mental health in the Caddo Parish Public School system in Northwest Louisiana at the time of the survey. This paper focuses on the effect of SM usage on body image. An exploratory analysis was performed based on this objective.

### Statistical analysis

Data management and statistical analysis were performed using STATA/BE v17 (StataCorp, LLC). Continuous variables are presented as mean standard deviation (SD). Categorical variables are presented as the number (proportion or %) of participants. All data were tested for normality using Kolmogorov-Smirnov test, and data that passed normality assumption were analyzed using Student’s *t*-test (*p* < 0.05) for two groups. Categorical variables were analyzed using Pearson’s Chi-squared test.

Unadjusted and adjusted association of gender and endpoints were analyzed using generalized logistic regression models for the entire dataset. All *P*-values are 2-sided, with *p* < 0.05 considered statistically significant.

## Results

### Participants

Out of the 11,248 total high school students in Caddo Parish Public School System, we sampled over 50% with a sample size of 5,070 (30). Respondents were predominately females (54.3%) with an average age of 15.79 ± 1.23 years.

### Hypothesis 1

In Hypothesis 1, we hypothesized that females are more likely to use SM than males.

We found that only 3.2% of sampled adolescents reported not using SM (*p* < 0.001) (Table 1). A greater percentage of females reported using Facebook, Instagram, Pinterest, Snapchat, and TikTok than males (*p* < 0.001) (Table 1). A greater percentage of females reported using Twitter and Instagram than males, but results were not statistically significant. The only SM application used by males more than females was Reddit (*p* < 0.001) (Table 1). Further, females were more likely to spend two or more hours on SM per day as compared to males (*p* < 0.001) (Table 1).

**TABLE 1:** Differences in social media (SM) use by sex.

	Total sample *N* = 5,070	Female *N* = 2,753	Male *N* = 2,203	*P*
**I do not use social media**				< 0.001
No	3,424 (67.5%)	2,064 (75.0%)	1,265 (57.4%)	
Yes	162 (3.2%)	63 (2.3%)	92 (4.2%)	
**Facebook**				< 0.001
No	2,756 (54.4%)	1,576 (57.2%)	1,100 (49.9%)	
Yes	830 (16.4%)	551 (20.0%)	257 (11.7%)	
**Instagram**				< 0.001
No	582 (11.5%)	269 (9.8%)	285 (12.9%)	
Yes	3,004 (59.3%)	1,858 (67.5%)	1,072 (48.7%)	
**Pinterest**				< 0.001
No	2,749 (54.2%)	1,390 (50.5%)	1,276 (57.9%)	
Yes	837 (16.5%)	737 (26.8%)	81 (3.7%)	
**Reddit**				< 0.001
No	3,242 (63.9%)	2,022 (73.4%)	1,137 (51.6%)	
Yes	344 (6.8%)	105 (3.8%)	220 (10.0%)	
**Snapchat**				< 0.001
No	1,142 (22.5%)	531 (19.3%)	562 (25.5%)	
Yes	2,444 (48.2%)	1,596 (58.0%)	795 (36.1%)	
**TikTok**				< 0.001
No	1,889 (37.3%)	908 (33.0%)	915 (41.5%)	
Yes	1,697 (33.5%)	1,219 (44.3%)	442 (20.1%)	
**Twitter**				0.97
No	2,587 (51.0%)	1,535 (55.8%)	980 (44.5%)	
Yes	999 (19.7%)	592 (21.5%)	377 (17.1%)	
**YouTube**				0.54
No	872 (17.2%)	512 (18.6%)	339 (15.4%)	
Yes	2,714 (53.5%)	1,615 (58.7%)	1,018 (46.2%)	
**Other social media**				< 0.001
No	3,386 (66.8%)	2,044 (74.2%)	1,256 (57.0%)	
Yes	200 (3.9%)	83 (3.0%)	101 (4.6%)	
**Number of social media platforms used, mean (SD)**	3.69 (1.85)	3.99 (1.76)	3.24 (1.86)	< 0.001
**Time spent on social media daily**				< 0.001
Less than 1 h	493 (9.7%)	222 (8.1%)	253 (11.5%)	
1–2 h	918 (18.1%)	525 (19.1%)	371 (16.8%)	
More than 2 h	2,135 (42.1%)	1,367 (49.7%)	706 (32.0%)	

### Hypothesis 2

In Hypothesis 2, we hypothesized that the more time spent on SM, the more likely females are to report BII, with males remaining largely unaffected.

We found that 77.6% of females reported BII compared to 18% of males (*p* < 0.001) (Table 2). Females were about four times more likely to report experiencing BII compared to male participants with an odds ratio (OR) of 4.03 (95% confidence interval (CI) = 3.41–4.78, *p* < 0.001) (Table 3).

**TABLE 2:** Differences in social media (SM) use by self-report of body image issues (BII).

	Total sample *N* = 5,070	No BII *N* = 2,448	BII *N* = 1,192	*P*
**Age (years), mean (SD)**	15.79 (1.23)	15.76 (1.22)	15.83 (1.22)	0.14
**Gender**				< 0.001
Female	2,753 (54.3%)	1,236 (50.5%)	925 (77.6%)	
Male	2,203 (43.5%)	1,159 (47.3%)	215 (18.0%)	
Other	114 (2.3%)	53 (2.2%)	52 (4.4%)	
**Do not use social media**				0.021
No	3,424 (67.5%)	2,252 (92.0%)	1,132 (95.0%)	
Yes	162 (3.2%)	122 (5.0%)	40 (3.4%)	
**Facebook**				0.037
No	2,756 (54.4%)	1,843 (75.3%)	873 (73.2%)	
Yes	830 (16.4%)	531 (21.7%)	299 (25.1%)	
**Instagram**				0.13
No	582 (11.5%)	378 (15.4%)	164 (13.8%)	
Yes	3,004 (59.3%)	1,996 (81.5%)	1,008 (84.6%)	
Missing	1,484 (29.3%)	74 (3.0%)	20 (1.7%)	
**Pinterest**				< 0.001
No	2,749 (54.2%)	1,959 (80.0%)	750 (62.9%)	
Yes	837 (16.5%)	415 (17.0%)	422 (35.4%)	
**Reddit**				< 0.001
No	3,242 (63.9%)	2,176 (88.9%)	1,026 (86.1%)	
Yes	344 (6.8%)	198 (8.1%)	146 (12.2%)	
**Snapchat**				< 0.001
No	1,142 (22.5%)	809 (33.0%)	293 (24.6%)	
Yes	2,444 (48.2%)	1,565 (63.9%)	879 (73.7%)	
**TikTok**				< 0.001
No	1,889 (37.3%)	1,373 (56.1%)	476 (39.9%)	
Yes	1,697 (33.5%)	1,001 (40.9%)	696 (58.4%)	
**Twitter**				< 0.001
No	2,587 (51.0%)	1,753 (71.6%)	794 (66.6%)	
Yes	999 (19.7%)	621 (25.4%)	378 (31.7%)	
**YouTube**				< 0.001
No	872 (17.2%)	613 (25.0%)	219 (18.4%)	
Yes	2,714 (53.5%)	1,761 (71.9%)	953 (79.9%)	
**Other social media**				< 0.001
No	3,386 (66.8%)	2,287 (93.4%)	1,099 (92.2%)	
Yes	200 (3.9%)	87 (3.6%)	73 (6.1%)	
**Number of social media platforms used, mean (SD)**	3.69 (1.85)	3.47 (1.76)	4.25 (1.87)	< 0.001
**Time spent on social media daily**				< 0.001
Less than 1 h	493 (9.7%)	385 (15.7%)	108 (9.1%)	
1– h	918 (18.1%)	645 (26.3%)	273 (22.9%)	
More than 2 h	2,135 (42.1%)	1,344 (54.9%)	791 (66.4%)	

**TABLE 3:** Odds ratios for self-reported body image issues (BII).

Predictors	OR	95% CI	*P*
**Female vs. male**	4.03	3.41–4.78	< 0.001
**Social media**			
No use	0.65	0.45–0.94	0.021
Facebook	1.19	1.01–1.40	0.038
Instagram	1.16	0.95–1.42	0.133
Pinterest	2.66	2.26–3.12	< 0.001
Reddit	1.56	1.25–1.96	< 0.001
Snapchat	1.55	1.33–1.81	< 0.001
TikTok	2.01	1.74–2.31	< 0.001
Twitter	1.34	1.15–1.57	< 0.001
YouTube	1.51	1.27–1.80	< 0.001
Other	1.75	1.27–2.40	0.001
**Time spent on social media daily**			
Less than 2 h (Reference)	1	−	−
More than 2 h	1.59	1.37–1.84	< 0.001
**Number of social media platforms used**			
Less than 4 platforms (Reference)	1	−	−
More than 4 platforms	2.04	1.77–2.36	< 0.001

**FIGURE 1:**
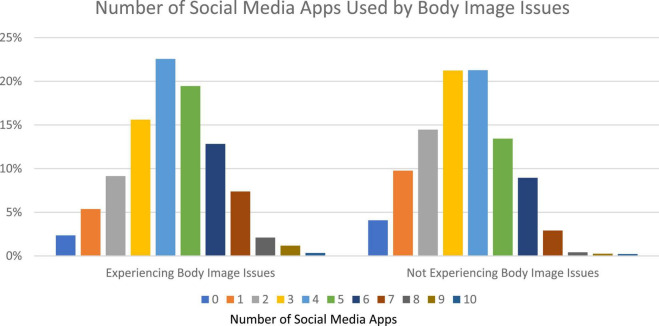
Distribution of the number of reported social media (SM) platforms used by respondents’ report of experiencing body image issues (BII).

**FIGURE 2:**
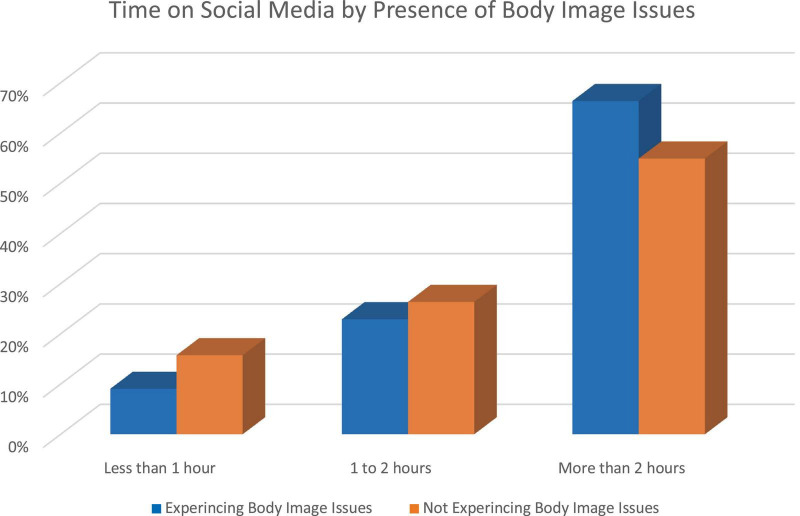
Increase in daily time spent on social media (SM) platforms has a dose-response relationship to increasing self-reported body image issues (BII).

Students reporting a daily use of more than 2 h of SM have 1.59 more odds of self-reporting BII compared to those using less than 2 h daily (CI = 1.37–1.84, *p* < 0.001). Students who reported use of more than four SM platforms have two times greater odds of reporting BII than do those using less than four of these platforms (OR = 2.04, CI = 1.77–2.36, *p* < 0.001) (Table 3). Most respondents used three or four SM apps (Figure 1), which corresponded to peak reports of BII (Table 3). Using five SM platforms increased self-reports of BII by about 30% and using six or seven platforms doubled reports of BII (Figure 1). Students who reported experiencing BII, were more likely to spend two or more hours a day on SM, as compared to those who did not report BII (Figure 2). After 2 h spent daily on SM platforms, BII reported in SM users increased from 23% of respondents to 66% of respondents. Contrary to our hypothesis, data showed both females and males to self-report BII in the setting of increased time using SM.

### Hypothesis 3

In Hypothesis 3 we hypothesized that HVSM platforms are associated with greater reports of BII than non-HVSM platforms.

Pinterest, Snapchat, TikTok, and YouTube show a statistically significant difference in self-reporting BII (35.4, 73.7, 58.4, and 79.9%, respectively, with *p* < 0.001). The use of non-HVSM Facebook, Reddit, and Twitter also showed to be associated with self-reported BII (25.1%, *p* = 0.037; 12.2%, *p* < 0.001; 31.7%, *p* < 0.001, respectively). Use of Instagram does not show a statistically significant difference in self-reported BII with usage (84.6%, *p* = 0.13) (Table 2).

Pinterest showed the highest odds of self-reporting BII (OR = 2.66, 95% CI = 2.26–3.12, *p* < 0.001) followed by TikTok (OR = 2.01, 95% CI = 1.74–2.31, *p* < 0.001), Snapchat (OR = 1.55, 95% CI = 1.33–1.81, *p* < 0.001) and YouTube (OR = 1.51, 95% CI = 1.27–1.80, *p* < 0.001) (Table 3). Non-HVSM of Facebook and Twitter showed less odds of self-reporting BII while still remaining statistically significant (OR = 1.19, 95% CI = 1.01–1.40, *p* = 0.038; OR = 1.34, 95% CI = 1.15–1.57, *p* < 0.001). The use of Instagram was not statistically significant with self-reporting of BII in this sample of respondents (OR = 1.16; 95% CI = 0.95–1.42; *p* = 0.133). Not using SM was protective for self-reporting BII (OR = 0.65, 95% CI = 0.45–0.94, *p* < 0.021) (Table 3).

When stratified by sex, HVSM predictors showed increased odds of reporting BII in both females and males who use Pinterest (OR = 1.66, 95% CI = 1.38–1.99, *P* < 0.001 and OR = 3.31, 95% CI = 2.05–5.36, *P* < 0.001), YouTube (OR = 1.61, 95% CI = 1.31–1.99, *P* < 0.001 and OR = 1.51, 95% CI = 1.03–2.21, *P* = 0.033), and TikTok (OR = 1.65, 95% CI = 1.38–1.97, *P* < 0.001 and OR = 1.50, 95% CI = 1.11–2.03, *P* < 0.01). Snapchat was statistically significant for females (OR = 1.30, 95% CI = 1.06–1.59, *P* < 0.05) but not males (OR = 1.31, 95% CI = 0.96–1.78, *P* = 0.09) in association with BII reporting (Table 4). While the use of HVSM was more likely to result in reports of BII, our data was unable to make an association between HVSM and increased BII. A diversity of SM platforms (both HVSM and non-HVSM) were associated with a statistically significant increase in BII among participants.

**TABLE 4:** Odds ratios for self-reporting body image issues (BII) stratified by sex for highly visual social media (HVSM).

Predictors	OR	95% CI	*P*
**Females**			
Instagram	1.05	0.80 to 1.36	0.730
Pinterest	1.66	1.38 to 1.99	<0.001
YouTube	1.61	1.31 to 1.99	<0.001
Snapchat	1.30	1.06 to 1.59	<0.05
TikTok	1.65	1.38 to 1.97	<0.001
**Males**			
Instagram	0.88	0.61 to 1.27	0.510
Pinterest	3.31	2.05 to 5.36	< 0.001
YouTube	1.51	1.03 to 2.21	0.033
Snapchat	1.31	0.96 to 1.78	0.09
TikTok	1.50	1.11 to 2.03	<0.01

**FIGURE 3:**
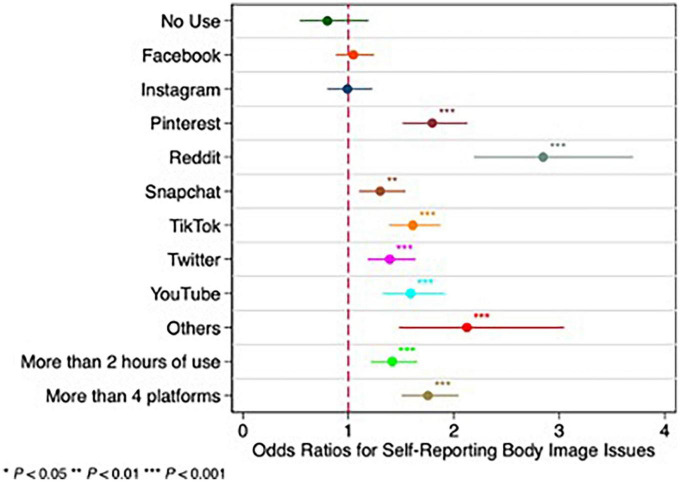
Odds ratios for self-reporting body image issues (BII) after adjusting for sex.

All predictors remained statistically significant after adjustment for sex differences, apart from “No use of SM” (OR = 0.80; 95% CI = 0.54–1.19; *p* = 0.27) and Facebook (OR = 1.05, 95% CI = 0.88–1.24; *p* = 0.617). Pinterest and Reddit were among the strongest contributors to BII, with OR = 1.79 (CI = 1.51–2.13; *p* < 0.001) and OR = 2.85 (CI = 2.19–3.70; *p* < 0.00), respectively. Students spending 2 h or more on SM had increased odds of self-reporting BII regardless of sex differences (Figure 3). Males were over two times as likely to use Reddit as compared to females (10% of the users were male vs. 3.8% of the users who were female). Although Reddit showed a statistically significant contribution to self-reporting BII (OR = 2.85; *p* < 0.001), the CI was large at 2.19–3.70, which may be attributed to most participants not using Reddit; regardless, those who do use the platform exhibit self-reporting BII. Data show that Reddit’s contribution to BII is significant but may not be meaningful due to the low percentage of users in the study population.

## Discussion

A tremendous gap in the literature exists in studying populations of the highest index of social vulnerability and health disparities regarding SM use and BII. There is a robust database of literature on other populations, including Latinx, Chinese, British, Australian, young adult, and university populations. This is the only study to date that has assessed children, majority Americans of African descent, of the highest percentile of social vulnerability across an entire parish (47–50). No previous studies have looked at impoverished adolescents with a sample size of such magnitude; others of similar size focus on 18 years and older or neglect to account for social minority populations (50, 52–54). The current study examined whether self-reported BII was associated with SM use among 5,070 high school adolescents in Northwest Louisiana.

### Social media platform usage: Male vs. females

One of the strongest drivers of BII is the reception of appearance-related feedback on SM (56). Although both males and females may be negatively impacted by SM, Manago et al., argues that females may receive a disproportionate amount of peer appearance-related feedback than males, with their data revealing females are evaluated more strongly on SM than males regarding their appearances (57). Following this, most research available to date has thoroughly explored the relationship between SM and the development of BII in females, with the studies that did look at both males and females emphasizing the latter. This includes a large body of literature dedicated to eating disorders (58–64). McCabe and Ricciardelli, have demonstrated overlap between the factors that affect both females and males in the development of BII, highlighting the importance of SM and BII studies that sample both males and females (65).

In contrast with many previous studies, our study sampled both females and males. Findings echo previous data in the disproportionate disparities of BII faced by females as compared to males with 77.6% females vs. nearly 20% of males reporting BII. Despite these similarities, researchers highlight a vulnerable population that, alongside similar BII, faces tremendous health disparities significantly higher than national averages. The public health crisis in Louisiana arguably leaves a wider gap in the equitable care necessary to rehabilitate the vulnerable adolescent population from BII and associated mental health disparities. Caddo Parish is one of the poorest parishes in Louisiana in a state that ranks 49th out of 50 in mental healthcare and mental health outcomes (34, 40).

### Specific social media platforms related to body image issues

A large percentage of existing data on SM and body image reports findings in terms of Facebook usage, given its nearly three billion users (42, 61, 66–68). Previous studies have included SM variables of Facebook, Pinterest, Reddit, TikTok, and YouTube in conglomerate with results expressed in total SM usage and time (69). Other studies have collected robust data on the influence of solely Instagram and Snapchat contributing to BII in both males and females (70–72). In comparison, our data presents novel findings across many different SM platforms (Instagram, Facebook, Snapchat, TikTok, Pinterest, YouTube, Reddit, and Twitter) by exploring the relationship of BII and SM through assessment of specific SM platforms used by males vs. females and their individual influences on BII. Findings indicated that females were more likely to use Pinterest, TikTok, Instagram, Snapchat, and Twitter; males were more likely to use Reddit. These findings are supported by past research showing females more likely to use SM as compared to males (17, 42, 64). Pinterest was the platform most highly associated with BII in Caddo respondents with usage for two or more hours a day resulting in 2.66 greater odds of developing BII.

Of note, Reddit revealed significantly higher odds of association with BII than other platforms. Reddit is a diverse platform that can both contribute to negative perception of self as well as operate as an outlet for individuals facing BII to commiserate and collectively soothe pain (73). Many sources reveal Reddit may offer a support community for various struggles from eating disorders to image-based dissatisfaction, while also at times endorsing and empowering negative self-perception, eating disorders, and BII (74–76). During times of isolation during the COVID-19 pandemic, Reddit has been shown as a space for reprieve from loneliness (77). These data reveal Reddit has potential to be both beneficial and harmful regarding BII (78). From our data, it is unclear if Reddit use is contributing to BII, or if Reddit is merely a coping outlet for those already experiencing BII; therefore, we cannot make assumptions on the direction of causality between Reddit and BII. This was an unexpected finding and should be investigated in future research.

While the use of HVSM was more likely to result in reports of BII, our data was unable to make an association between HVSM and increased BII. A diversity of SM platforms (both HVSM and non-HVSM) were associated with a statistically significant increase in BII among participants. Our results highlight a frightening reality of mental disease and BII for the majority of adolescents who use SM in this community; a community comprised of a majority of Americans of African descent, a population who, along with adolescents of Hispanic/Latinx descent, have been shown to use up to 2 h more screen time daily as compared to their European-descended counterparts (15).

### Time on platforms related to development of body image issues

Previous data has shown adolescents of African descent and socioeconomically vulnerable populations average up to 36% more SM use and screen time than adolescents of European descent and higher income families (15). Looking at the adolescent community of Caddo Parish, a community comprised of majority Americans of African descent, data revealed over 60% of participants experiencing BII spent in excess of two or more hours a day on SM. This finding echoes that of numerous other sources who show similarly that time is positively associated with development of and persistence of BII (62, 79–81). Vandenbosch and Eggermont, found a positive correlation between frequency of checking SM accounts and increased body surveillance (81). de Vries et al., further found that the frequency of checking Facebook translated to increased investment in adolescent appearance (82). This dose-response relationship is also validated by several studies that found body dissatisfaction increases with total time spent on SM (83, 84).

Contrary to our hypothesis, data showed both females and males to self-report BII in the setting of increased time using SM; this is concordant with previous studies. A dose-response relationship between SM use and BII indicates BII and associated mental health issues transcend culture and social vulnerability (85, 86). Despite this, data emphasizes that outcomes have tremendous potential for variability given that vulnerable populations may not have the resources to mitigate the deleterious effects of negative self-perception and BII that other, non-impoverished communities may have access to.

### Quantity of platforms related to body image issues

Several studies exist that focus solely on the SM platforms of Instagram, Facebook, or the combination; most data reported SM use generally associated with development of BII (11, 22, 26, 42, 61). Further, most data expressed outcomes in terms of SM use and BII but fail to individuate the discrete platforms’ effect on BII (80, 87, 88). Our data revealed statistically significant associations between SM use and BII for seven of eight distinct platforms sampled: Facebook, Pinterest, Reddit, Snapchat, TikTok, Twitter, and YouTube; no statistically significant values for Instagram were shown to be associated with development of BII. Our findings emphasize that a diversity of mediums and content seem to broadly have negative impacts on body image.

Results further indicated concomitant use of three to four SM platforms to correlate with the highest burden of BII suffered. This is thought to be due, at least in part, to the low number of respondents who selected more or fewer platforms on the survey. Other studies have shown a positive correlation between increasing SM platform usage and the development of BII thoughts and habits, such as disordered eating (61, 89).

Findings in this impoverished adolescent population parallel that of other studies, showing that the association of SM and BII have a broadly bad effect on adolescent wellbeing. Regardless, these similarities are not determinant of long-term outcomes. The tremendous health disparities faced by these vulnerable communities has potential to impede recovery and perpetuate deleterious mental health insults, such as BII.

### Potential limitations

There are limitations associated with the present study. Data were collected from a self-reported survey using a non-validated survey instrument; the questions were written by a team of dedicated adolescent peers and then edited by Step Forward team members. This study also neglected to assess race as a determinant of BII and general health; these design elements and measures will be included in future studies. This study only sampled a single population and may be cause for non-generalizability of results when applying to broader populations outside of Northwest Louisiana. This study also did not utilize formal tools for measuring SM usage; an objective form of measuring SM activity and engagement may be beneficial for future studies. Finally, this study was conducted prior to the COVID-19 outbreak, so results may not accurately reflect the current mental landscape of Northwest Louisiana youth. It is a concern that adolescent BII and self-perception may be worse than depicted (90–92). Despite the limitations in the study design, we believe the study maintains clinical significance by clearly presenting the cogent relationship between SM usage and BII in a population devastated by health disparities and social vulnerability; the abundance of literature cited links BII to concurrent or later development of conditions that include, but are not limited to, eating disorders, suicidality, and general mental health disturbances such as anxiety and depression. We also recognize that the classification of BII and criteria for inclusion of this as a variable may be perceived as vague in the study design. The instrument designers felt it important to provide respondents with terms that were familiar with or would readily understand when answering the survey. With this in mind, more generalizable terms such as “issues with body image” were employed. This phrase was used to capture the landscape of adolescent BII more broadly and to “rule in” respondents who may be affected rather than create more narrow, specific criteria that have the potential to “rule out” due to respondent incomprehension or non-identification with an unfamiliar language. Regardless of whether these terms meet specific clinical definitions, we believe the consistent use of student self-reporting throughout the survey provides sufficient indication of student perceived harm for us to warrant study.

### Future directions

Adolescent mental health disorders represent a serious problem and a significant burden of disease to the population. As adolescents increasingly spend more time on SM, such as they have since the COVID pandemic, the risk of related harms, such as BII, may increase as well.

This study produced valuable data on particularly vulnerable populations that may prove relevant and useful in highlighting BII and mental health challenges among those who face extreme health disparities and social vulnerability. Our data echoes current literature in that SM use is associated with significant BII in adolescents. However, no other study has assessed a population wherein the preponderance of children across an entire parish that is majority Americans of African descent or that ranks in the highest percentile for social vulnerability (47–50). The consistency of our data with previous findings demonstrates the relationship between SM use and BII to transcend that of culture and socioeconomic status and be broadly deleterious for the adolescent population, a finding that would not be otherwise known until now. The current public health crisis in Louisiana highlights a gap in equitable care for these populations, a gap that may selectively impede recovery from BII and associated mental health disparities. Given the plasticity and sensitivity of the adolescent brain, inaction during this mental health crisis may have devastating effects on the lives of countless of adolescents (93).

Our research stresses the importance of understanding the factors affecting the health and wellbeing of this vulnerable adolescent community. Obtaining data from the lived experiences of adolescents may help researchers better understand and address these mental health crises. Furthermore, we believe direct partnership with the adolescent community may be essential for development of future supportive interventions. Findings may correspondingly suggest that platform providers prioritize development of tools to reduce harmful body image content available to vulnerable adolescents to mitigate the damaging effects of BII on adolescent present and future wellbeing.

## Data availability statement

The raw data supporting the conclusions of this article will be made available by the authors, without undue reservation.

## Ethics statement

The studies involving human participants were reviewed and approved by the Institutional Review Board and LSU Health Shreveport. Written informed consent to participate in this study was provided by the participants’ legal guardian/next of kin.

## Author contributions

CS: conceptualization, roles/writing—original draft, writing—review and editing, and submission. JM: data curation, conceptualization, roles/writing—original draft, and writing—review and editing. JT and RL: conceptualization, data curation, investigation, methodology, and writing—review and editing. TM: conceptualization and writing—review and editing. VA-Q and MB: formal analysis and writing—review and editing. PM and LA: data curation and writing—review and editing. JP: conceptualization, project administration, and writing—review and editing. KM: data curation, investigation, project administration, supervision, and writing—review and editing. All authors contributed to the article and approved the submitted version.

## References

[1] WHO. *Child and Adolescent Mental Health.* Geneva: World Health Organization (2016).

[2] Kessler RC Wai TC Demler O Walters EE. Prevalence, severity, and comorbidity of 12-month DSM-IV disorders in the National Comorbidity Survey Replication. *Arch Gen Psychiatry.* (2005) 62:617–27. 10.1001/archpsyc.62.6.617 15939839 PMC2847357

[3] Colizzi M Lasalvia A Ruggeri M. Prevention and early intervention in youth mental health: is it time for a multidisciplinary and trans-diagnostic model for care? *Int J Ment Health Syst.* (2020) 14:1–14. 10.1186/s13033-020-00356-9 32226481 PMC7092613

[4] Merikangas KR He JP Burstein M Swanson SA Avenevoli S Cui L Lifetime prevalence of mental disorders in US adolescents: results from the national comorbidity study-adolescent supplement (NCS-A). *J Am Acad Child Adolesc Psychiatry.* (2010) 49:980–9. 10.1016/j.jaac.2010.05.017 20855043 PMC2946114

[5] Cash TF. Body image: past, present, and future. *Body Image.* (2004) 1:1–5. 10.1016/S1740-1445(03)00011-1 18089136

[6] Fouts G Burggraf K. Television situation comedies: female body images and verbal reinforcements. *Sex Roles.* (1999) 40:473–81. 10.1023/A:1018875711082

[7] Al Sabbah H Vereecken CA Elgar FJ Nansel T Aasvee K Abdeen Z Body weight dissatisfaction and communication with parents among adolescents in 24 countries: international cross-sectional survey. *BMC Public Health.* (2009) 9:52. 10.1186/1471-2458-9-52 19200369 PMC2645388

[8] Betz DE Ramsey LR. Should women be “all about that bass?”: diverse body-ideal messages and women’s body image. *Body Image.* (2017) 22:18–31. 10.1016/j.bodyim.2017.04.004 28554090

[9] Betz DE Sabik NJ Ramsey LR. Ideal comparisons: body ideals harm women’s body image through social comparison. *Body Image.* (2019) 29:100–9. 10.1016/j.bodyim.2019.03.004 30901739

[10] Clayton RB Ridgway JL Hendrickse J. Is plus size equal? The positive impact of average and plus-sized media fashion models on women’s cognitive resource allocation, social comparisons, and body satisfaction. *Commun Monogr.* (2017) 84:406–22. 10.1080/03637751.2017.1332770

[11] Cohen R Fardouly J Newton-John T Slater A. #BoPo on Instagram: an experimental investigation of the effects of viewing body positive content on young women’s mood and body image. *New Media Soc.* (2019) 21:1546–64. 10.1177/1461444819826530

[12] Nagata JM Murray SB Bibbins-domingo K Garber AK Mitchison D Griffiths S Predictors of muscularity oriented disordered eating behaviors in U.S. young adults: a prospective cohort study. *Int J Eat Disord.* (2020) 52:1380–8. 10.1002/eat.23094 31220361 PMC6901753

[13] Robb MB. *Teens and the News: The Influencers, Celebrities, and Platforms They Say Matter Most, 2020.* San Francisco, CA: Common Sense Media (2020).

[14] Rideout Robb MBV. *The Common Sense Census: Media Use by Tweens and Teens, 2019.* San Francisco, CA: Common Sense Media (2019).

[15] Rideout V Peebles A Mann S Robb MB. *The Common Sense Census: Media Use by Tweens and Teens, 2021.* San Francisco, CA: Common Sense Media (2021).

[16] McCrory A Best P Maddock A. “It’s just one big vicious circle”: young people’s experiences of highly visual social media and their mental health. *Health Educ Res.* (2022) 37:167–84. 10.1093/her/cyac010 35543267

[17] Marengo D Longobardi C Fabris MA Settanni M. Highly-visual social media and internalizing symptoms in adolescence: the mediating role of body image concerns. *Comput Hum Behav.* (2018) 82:63–9. 10.1016/j.chb.2018.01.003

[18] Aspinal G. *“I Was Obsessed With Faceune”: 71% Of People Won’t Post A Picture Online Without Photoshopping It - That Needs To Change.* Milan: Grazia (2020).

[19] Griffiths S Murray SB Krug I McLean SA. The contribution of social media to body dissatisfaction, eating disorder symptoms, and anabolic steroid use among sexual minority men. *Cyberpsychol Behav Soc Netw.* (2018) 21:149–56. 10.1089/cyber.2017.0375 29363993 PMC5865626

[20] McBride C Costello N Ambwani S Wilhite B Austin SB. Digital manipulation of images of models’ appearance in advertising: strategies for action through law and corporate social responsibility incentives to protect public health. *Am J Law Med.* (2019) 45:7–31. 10.1177/0098858819849990 31293209

[21] Mahon C Hevey D. Processing body image on social media: gender differences in adolescent boys’ and girls’ agency and active coping. *Front Psychol.* (2021) 12:626763. 10.3389/fpsyg.2021.626763 34093311 PMC8175666

[22] Fioravanti G Tonioni C Casale S. #Fitspiration on Instagram: the effects of fitness−related images on women’s self−perceived sexual attractiveness. *Scand J Psychol.* (2021) 62:746–51. 10.1111/sjop.12752 34170526 PMC8518738

[23] Bucchianeri MM Fernandes N Loth K Hannan PJ Eisenberg ME Neumark-Sztainer D. Body dissatisfaction: do associations with disordered eating and psychological well-being differ across race/ethnicity in adolescent girls and boys? *Cultur Divers Ethnic Minor Psychol.* (2016) 22:137–46. 10.1037/cdp0000036 26052976 PMC6712990

[24] Robinson L Prichard I Nikolaidis A Drummond C Drummond M Tiggemann M. Idealised media images: the effect of fitspiration imagery on body satisfaction and exercise behaviour. *Body Image.* (2017) 22:65–71. 10.1016/j.bodyim.2017.06.001 28654826

[25] Brunborg GS Burdzovic Andreas J. Increase in time spent on social media is associated with modest increase in depression, conduct problems, and episodic heavy drinking. *J Adolesc.* (2019) 74:201–9. 10.1016/j.adolescence.2019.06.013 31254779

[26] Fardouly J Vartanian LR. Negative comparisons about one’s appearance mediate the relationship between Facebook usage and body image concerns. *Body Image.* (2015) 12:82–8. 10.1016/j.bodyim.2014.10.004 25462886

[27] Census US. *QuickFacts.* Caddo Parish, LA: Census US (2021).

[28] Louisiana Department of Education. *Caddo Parish Snapshot.* Baton Rouge, LA: Louisiana Department of Education (2020).

[29] U.S. News. *Overview of Caddo Parish, LA.* Caddo Parish, LA: U.S. News (2019).

[30] Public School Review,. *Caddo Parish School District* (2021). Available online at: https://www.publicschoolreview.com/louisiana/caddo-parish-school-district/2200300-school-district (accessed June, 2022).

[31] Greatschools. *School District Summary Ratings* (2022). Available online at: https://www.greatschools.org/louisiana/shreveport/caddo-parish-school-district/ (accessed June, 2022).

[32] National Center for Education Statistics. *Education Demographic and Geographic Estimates.* Washington, DC: National Center for Education Statistics (2021).

[33] Economic Research Service, U.S. Department of Agriculture. *Poverty.* Washington, DC: Economic Research Service, U.S. Department of Agriculture (2022).

[34] United Health Foundation. *2018 Annual Report.* Metairie, LA: United Health Foundation (2021).

[35] Norris DrDN, Norris DrAM. *2020 Community Counts.* Shreveport, LA: Community Foundation of North Louisiana (2020).

[36] Willis-Knighton Health System. *Bossier & Caddo Parish Community Health Needs Assessment.* Shreveport, LA: Willis-Knighton Health System (2019).

[37] Louisiana Department of Health. *Adult Obesity. Environmental Public Health Tracking.* Baton Rouge, LA: Louisiana Department of Health (2017).

[38] Gooch K. *10 Best, Worst States for Healthcare.* Chicago, IL: Becker’s Hospital Review (2021).

[39] McCann A. *Most Overweight and Obese Cities in the U.S. WalletHub.* (2022). Available online at: https://wallethub.com/edu/fattest-cities-in-america/10532 (accessed June, 2022).

[40] U.S. News. *Public Health Rankings.* Washington, DC: U.S. News (2021).

[41] CAHMI State Fact Sheet. *The Child and Adolescent Health Measurement Initiative*. Baltimore, MD: Child and Adolescent Health Measurement Initiative, Johns Hopkins Bloomberg School of Public Health (2019).

[42] Manago AM Monique Ward L Lemm KM Reed L Seabrook R. Facebook involvement, objectified body consciousness, body shame, and sexual assertiveness in college women and men. *Sex Roles.* (2014) 72:1–14. 10.1007/s11199-014-0441-1

[43] Sagrera CE Alderman LJ Goeders NE Murnane KS. Elucidating the role of trauma and significant life stress in the disease of addiction may provide new targets for medication development. *CNS Neurol Disord Drug Targets.* (2022) 22. 10.2174/1871527321666220511145230 [Epub ahead of print]. 35546748

[44] Spinazzola J Hodgdon HI Liang LJ Ford JD Layne CM Pynoos R Unseen wounds: the contribution of psychological maltreatment to child and adolescent mental health and risk outcomes. *Psychol Trauma Theory Res Pract Policy.* (2014) 6:S18–28. 10.1037/a0037766

[45] Lippard ETC Nemeroff CB. The devastating clinical consequences of child abuse and neglect: increased disease vulnerability and poor treatment response in mood disorders. *Am J Psychiatry.* (2020) 177:20–36. 10.1176/appi.ajp.2019.19010020 31537091 PMC6939135

[46] Cecil J Picard Center for Child Development and Lifelong Learning. *2020 Louisiana Caring Communities Youth Survey.* (2021). Available online at: https://picardcenter.louisiana.edu/sites/picardcenter/files/2020%20Parish%20Reports_Caddo%20Parish520Profile%20Report.pdf (accessed September 11, 2021).

[47] Lev-Ari L Baumgarten-Katz I Zohar AH. Mirror, mirror on the wall: how women learn body dissatisfaction. *Eat Behav.* (2014) 15:397–402. 10.1016/j.eatbeh.2014.04.015 25064289

[48] Bakshi A Van Doren A Maser C Aubin K Stewart C Soileau S Identifying Louisiana communities at the crossroads of environmental and social vulnerability, COVID-19, and asthma. *PLoS One.* (2022) 17:e0264336. 10.1371/journal.pone.0264336 35196332 PMC8865632

[49] Halliwell E Easun A Harcourt D. Body dissatisfaction: can a short media literacy message reduce negative media exposure effects amongst adolescent girls?: short media literacy message. *Br J Health Psychol.* (2011) 16:396–403. 10.1348/135910710X515714 21489065

[50] McCabe MP Ricciardelli LA Holt K. Are there different sociocultural influences on body image and body change strategies for overweight adolescent boys and girls? *Eat Behav.* (2010) 11:156–63. 10.1016/j.eatbeh.2010.01.005 20434062

[51] Niche. *Caddo Parish Public Schools.* Pittsburgh, PA: Niche (2022).

[52] Barthorpe A Winstone L Mars B Moran P. Is social media screen time really associated with poor adolescent mental health? A time use diary study. *J Affect Disord.* (2020) 274:864–70. 10.1016/j.jad.2020.05.106 32664027

[53] Gordon CS Jarman HK Rodgers RF McLean SA Slater A Fuller-Tyszkiewicz M Outcomes of a cluster randomized controlled trial of the SoMe social media literacy program for improving body image-related outcomes in adolescent boys and girls. *Nutrients.* (2021) 13:3825. 10.3390/nu13113825 34836084 PMC8674763

[54] Burnette CB Kwitowski MA Mazzeo SE. “I don’t need people to tell me I’m pretty on social media:” A qualitative study of social media and body image in early adolescent girls. *Body Image.* (2017) 23:114–25. 10.1016/j.bodyim.2017.09.001 28965052

[55] Student Attributes. *October 2019 Student Attributes by Site and by School System.* (2019). Available online at: https://www.louisianabelieves.com/resources/library/student-attributes (accessed June, 2022).

[56] Clark L Tiggemann M. Appearance culture in nine- to 12-year-old girls: media and peer influences on body dissatisfaction. *Soc Dev.* (2006) 15:628–43. 10.1111/j.1467-9507.2006.00361.x

[57] Manago AM Graham MB Greenfield PM Salimkhan G. Self-presentation and gender on MySpace. *J Appl Dev Psychol.* (2008) 29:446–58. 10.1016/j.appdev.2008.07.001

[58] Morris AM Katzman DK. The impact of the media on eating disorders in children and adolescents. *Paediatr Child Health.* (2003) 8:287–9. 10.1093/pch/8.5.287 20020030 PMC2792687

[59] Latzer Y Spivak-Lavi Z Katz R. Disordered eating and media exposure among adolescent girls: the role of parental involvement and sense of empowerment. *Int J Adolesc Youth.* (2015) 20:375–91. 10.1080/02673843.2015.1014925

[60] McCabe MP Ricciardelli LA. Parent, peer, and media influences on body image and strategies to both increase and decrease body size among adolescent boys and girls. *Adolescence.* (2001) 36:225–40. 11572302

[61] Hummel AC Smith AR. Ask and you shall receive: desire and receipt of feedback via Facebook predicts disordered eating concerns. *Int J Eat Disord.* (2015) 48:436–42. 10.1002/eat.22336 25060558

[62] Klein KM. Why don’ t I look like her? The impact of social media on female body image. *Claremont Coll.* (2013) 27:1–124.

[63] Puglia DR. *Social Media Use and Its Impact on Body Image: The Effects of Body Comparison Tendency, Motivation for Social Media Use, and Social Media Platform on Body Esteem in Young Women.* Chapel Hill, NC: University of North Carolina at Chapel Hill Graduate School (2017).

[64] Hogue JV Mills JS. The effects of active social media engagement with peers on body image in young women. *Body Image.* (2019) 28:1–5. 10.1016/j.bodyim.2018.11.002 30439560

[65] McCabe MP Ricciardelli LA. Body image dissatisfaction among males across the lifespan: a review of past literature. *J Psychosom Res.* (2004) 56:675–85. 10.1016/S0022-3999(03)00129-6 15193964

[66] Statista Research Department. *Facebook: Number of Monthly Active Users Worldwide 2008-2021.* Hamburg: Statista Research Department (2021).

[67] Kim JW Chock MT. Body image 2.0: associations between social grooming on Facebook and body image concerns. *Comput Hum Behav.* (2015) 48:331–9. 10.1016/j.chb.2015.01.009

[68] Tiggemann M Slater A. NetTweens: the internet and body image concerns in preteenage girls. *J Early Adolesc.* (2014) 34:606–20. 10.1177/0272431613501083

[69] Sidani JE Shensa A Hoffman B Hanmer J Primack BA. The association between social media use and eating concerns among U.S. young adults. *J Acad Nutr Diet.* (2016) 116:1465–72. 10.1016/j.jand.2016.03.021 27161027 PMC5003636

[70] Burnell K Kurup AR Underwood MK. Snapchat lenses and body image concerns. *New Media Soc.* (2021) 24:2088-106. 10.1177/1461444821993038

[71] Tiggemann M Anderberg I. Social media is not real: the effect of ‘Instagram vs. reality’ images on women’s social comparison and body image. *New Media Soc.* (2020) 22:2183–99. 10.1177/1461444819888720

[72] Chatzopoulou E Filieri R Dogruyol SA. Instagram and body image: motivation to conform to the “Instabod” and consequences on young male wellbeing. *J Consum Aff.* (2020) 54:1270–97. 10.1111/joca.12329

[73] Signorelli J. Of pumpkin spice lattes and hamplanets the venting genre as support and subversion on Reddit’s r/FatPeopleStories. *J Lang Aggress Confict.* (2021) 10:64–84. 10.1075/jlac.00067.sig 33486653

[74] Andy A. Studying how individuals who express the feeling of loneliness in an online loneliness forum communicate in a nonloneliness forum: observational study. *JMIR Form Res.* (2021) 5:e28738. 10.2196/28738 34283026 PMC8335613

[75] Miller B. Investigating reddit self-disclosure and confessions in relation to connectedness, social support, and life satisfaction. *Soc Media Soc.* (2020) 9:39–62.

[76] Sowles SJ McLeary M Optican A Cahn E Krauss MJ Fitzsimmons-Craft EE A content analysis of an online pro-eating disorder community on Reddit. *Body Image.* (2018) 24:137–44. 10.1016/j.bodyim.2018.01.001 29414146 PMC5869127

[77] Nutley S Falise A Henderson R Apostolou V Mathews C Striley C. Impact of the COVID-19 pandemic on disordered eating behavior: qualitative analysis of social media posts. *JMIR Ment Health.* (2021) 8:e26011. 10.2196/26011 33465035 PMC7842857

[78] Thorstad R Wolff P. Predicting future mental illness from social media: a big-data approach. *Behav Res Methods.* (2019) 51:1586–600. 10.3758/s13428-019-01235-z 31037606

[79] Tsitsia AK Tzavela EC Janikian M Olafsson K Iordache A Scoenmakers TM Online social networking in adolescence: patterns of use in six European countries and links with psychosocial functioning. *J Adolesc Health.* (2014) 55:141–7. 10.1016/j.jadohealth.2013.11.010 24618179

[80] Jiotsa B Naccache B Duval M Rocher B Grall-Bronnec M. Social media use and body image disorders: association between frequency of comparing one’s own physical appearance to that of people being followed on social media and body dissatisfaction and drive for thinness. *Int J Environ Res Public Health.* (2021) 18:1–14. 10.3390/ijerph18062880 33799804 PMC8001450

[81] Vandenbosch L Eggermont S. Understanding sexual objectification: a comprehensive approach toward media exposure and girls’ internalization of beauty ideals, self-objectification, and body surveillance. *J Commun.* (2012) 62:869–87. 10.1111/j.1460-2466.2012.01667.x

[82] de Vries DA Peter J Nikken P de Graaf H. The effect of social network site use on appearance investment and desire for cosmetic surgery among adolescent boys and girls. *Sex Roles.* (2014) 71:283–95. 10.1007/s11199-014-0412-6

[83] De Vries DA Peter J De Graaf H Nikken P. Adolescents’ social network site use, peer appearance-related feedback, and body dissatisfaction: testing a mediation model. *J Youth Adolesc.* (2016) 45:211–24. 10.1007/s10964-015-0266-4 25788122 PMC4698286

[84] Twenge JM Martin GN. Gender differences in associations between digital media use and psychological well-being: evidence from three large datasets. *J Adolesc.* (2020) 79:91–102. 10.1016/j.adolescence.2019.12.018 31926450

[85] Rodgers RF Slater A Gordon CS McLean SA Jarman HK Paxton SJA. Biopsychosocial model of social media use and body image concerns, disordered eating, and muscle-building behaviors among adolescent girls and boys. *J Youth Adolesc.* (2020) 49:399–409. 10.1007/s10964-019-01190-0 31907699

[86] Yang H Wang JJ Tng GYQ Yang S. Effects of social media and smartphone use on body esteem in female adolescents: testing a cognitive and affective model. *Children.* (2020) 7:148. 10.3390/children7090148 32967376 PMC7552652

[87] Aparicio-Martinez P Perea-Moreno AJ Martinez-Jimenez MP Redel-Macías MD Pagliari C Vaquero-Abellan M. Social Media, thin-ideal, body dissatisfaction and disordered eating attitudes: an exploratory analysis. *Int J Environ Res Public Health.* (2019) 16:4177. 10.3390/ijerph16214177 31671857 PMC6861923

[88] Dumas AA Desroches S. Women’s use of social media: what is the evidence about their impact on weight management and body image? *Curr Obes Rep.* (2019) 8:18–32. 10.1007/s13679-019-0324-4 30666619

[89] Wilksch SM O’Shea A Ho P Byrne S Wade TD. The relationship between social media use and disordered eating in young adolescents. *Int J Eat Disord.* (2020) 53:96–106. 10.1002/eat.23198 31797420

[90] Swami V Horne G Furnham A. COVID-19-related stress and anxiety are associated with negative body image in adults from the United Kingdom. *Personal Individ Differ.* (2021) 170:110426. 10.1016/j.paid.2020.110426 33046945 PMC7539826

[91] Vall-Roqué H Andrés A Saldaña C. The impact of COVID-19 lockdown on social network sites use, body image disturbances and self-esteem among adolescent and young women. *Prog Neuropsychopharmacol Biol Psychiatry.* (2021) 110:110293–110293. 10.1016/j.pnpbp.2021.110293 33662532 PMC8569938

[92] Quathamer N Joy P. Being in a queer time: exploring the influence of the COVID-19 pandemic on LGBTQ+ body image. *Nutr Diet.* (2021) 79: 400–10. 34337841 10.1111/1747-0080.12699 PMC8441880

[93] Black MM Walker SP Fernald CH Anderson CT DiGirolamo AM Lu C Advancing early childhood development: from science to scale 1. *Lancet.* (2017) 47:549–62.

